# Systemic Activation of Activin A Signaling Causes Chronic Kidney Disease-Mineral Bone Disorder

**DOI:** 10.3390/ijms19092490

**Published:** 2018-08-23

**Authors:** Toshifumi Sugatani

**Affiliations:** Department of Pediatrics, Washington University School of Medicine, 660 S. Euclid, St. Louis, MO 63110, USA; sugatani_t@wustl.edu; Tel.: +1-314-286-2863; Fax: +1-314-286-2894

**Keywords:** activin A, osteoclast, osteoblast, CKD-MBD, vascular calcification

## Abstract

The high cardiovascular mortality associated with chronic kidney disease (CKD) is caused in part by the CKD-mineral bone disorder (CKD-MBD) syndrome. The CKD-MBD consists of skeletal, vascular and cardiac pathology caused by metabolic derangements produced by kidney disease. The prevalence of osteopenia/osteoporosis resulting from the skeletal component of the CKD-MBD, renal osteodystrophy (ROD), in patients with CKD exceeds that of the general population and is a major public health concern. That CKD is associated with compromised bone health is widely accepted, yet the mechanisms underlying impaired bone metabolism in CKD are not fully understood. Therefore, clarification of the molecular mechanisms by which CKD produces ROD is of crucial significance. We have shown that activin A, a member of the transforming growth factor (TGF)-β super family, is an important positive regulator of receptor activator of nuclear factor kappa-B ligand (RANKL)-induced osteoclastogenesis with Smad-mediated signaling being crucial for inducing osteoclast development and function. Recently, we have demonstrated systemic activation of activin receptors and activin A levels in CKD mouse models, such as diabetic CKD and Alport (AL) syndrome. In these CKD mouse models, bone remodeling caused by increased osteoclast numbers and activated osteoclastic bone resorption was observed and treatment with an activin receptor ligand trap repaired CKD-induced-osteoclastic bone resorption and stimulated individual osteoblastic bone formation, irrespective of *parathyroid hormone* (PTH) elevation. These findings have opened a new field for exploring mechanisms of activin A-enhanced osteoclast formation and function in CKD. Activin A appears to be a strong candidate for CKD-induced high-turnover ROD. Therefore, the treatment with the decoy receptor for activin A might be a good candidate for treatment for CKD-induced osteopenia or osteoporosis, indicating that the new findings from in these studies will lead to the identification of novel therapeutic targets for CKD-related and osteopenia and osteoporosis in general. In this review, we describe the impact of CKD-induced Smad signaling in osteoclasts, osteoblasts and vascular cells in CKD.

## 1. Introduction

Activin A is a homodimer composed of inhibin βA-βA subunits that belongs to the pleiotrophic family of the TGF-β superfamily of cytokines [1,2]. Activin A was initially discovered related to its capacity to induce the release of follicle-stimulating hormone [1,2]. Activin A acts through the serine/threonine kinase pathway common to other TGF-β superfamily members. Activin A signals by binding with high affinity to activin receptor type llA (ActRllA) or less so to activin receptor type llB (ActRIIB) followed by the recruitment of the activin type lB receptor, known as the activin receptor-like kinase 4 (ALK4), which belongs to the serine-threonine kinase receptors and is the main type l receptor for activin A. ActRIIA is the primary ligand binding protein for activin A, as ActRIIA is able to bind ligands in the absence of ALK4. However, ActRIIA is unable to trigger signal transduction without forming a complex with ALK4. Phosphorylation of ALK4 activates Smad2/3 and forms a complex together with Smad4 that translocates to the nucleus to regulate gene expression. ALK4 contains an extracellular domain with a number of conformationally important cysteine residues, a transmembrane domain, GS domain and an intracellular serine-threonine kinase domain. The unique and highly conserved GS domain regulates kinase activity in a phosphorylation-dependent manner. ActRIIA and ActRIIB have an extracellular ligand binding domain, a single transmembrane domain, an intracellular serine/threonine kinase domain and a PDZ protein-binding consensus sequence at the COOH-terminus [3]. In addition to Smad-dependent activin A signaling pathway, other non-canonical or Smad-independent effectors have been demonstrated in several tissues [4,5,6,7]. Activin A activates the mitogen-activated protein kinase (MAPK) signaling pathway including extracellular signal-regulated kinase 1/2 (ERK 1/2), c-Jun N-terminal kinase and p38 under inflammatory conditions [4]. In addition, activin A production is enhanced by inflammatory cytokines such as interleukin-1 through MAP kinase signaling pathway [5,6,7]. Yet, the relationship between the activated receptor complexes and non-canonical transduction remains to be defined. Activin A is involved in multiple vital biological processes in development and homeostasis, such as regulation of embryogenesis [8], development of the reproductive system [9], maintenance of pluripotent stem cells [8], regulation of immune response [10,11], wound healing [12,13], development of limbs [14,15,16] and craniofacial development [17,18,19].

Bone is a dynamic tissue that is constantly being remodeled to maintain a healthy skeleton, which is crucial for the efficient and lifelong execution of important skeletal functions. Bone has some vital functions. For instance, it acts as a metabolic organ with major reserves of calcium and phosphate. There are two major types of bone, cortical and trabecular. In particular, trabecular bone provides strength and the majority of the metabolic function. Trabecular bone is the major site of bone remodeling. Bone remodeling occurs in specialized modeling units spread throughout the skeleton that is regulated by mature osteoclasts and osteoblasts controlled by a variety of factors, such as cytokines, chemokines, hormones and biochemical stimuli. Osteoclasts remove the damaged bone and osteoblasts replace the resorbed matrix and mineralize it. Skeletal fragility in osteoporosis is caused by an imbalance in bone remodeling favoring osteoclast activity and is a significant cause of morbidity and mortality worldwide [20].

The kidney plays a critical role in the regulation of bone development and metabolism because it is the major organ that regulates calcium and phosphate homeostasis, which are indispensable elements for bone mineralization and development [21]. ROD consists of the pathological abnormalities in the bone of patients with CKD and it is a component of the CKD-MBD syndrome which causes a high incidence of skeletal fractures and contributes to the high mortality rates associated with kidney diseases [21]. The prevalence of osteopenia/osteoporosis in CKD patients exceeds that of the general population and is a major public health concern in patients with CKD [22]. That CKD is closely associated with compromised bone health is widely accepted, yet the mechanisms underlying impaired bone metabolism in CKD are unclear. Moreover, given that excessive osteoclastic bone resorption rates are consistent in ROD and contribute to hyperphosphatemia with stimulation of heterotopic mineralization including vascular calcification that produce high mortality in patients with CKD, deciphering the molecular mechanisms by which CKD produces excessive osteoclastic development and bone resorption is of crucial significance.

Recently, we have demonstrated systemic activation of ActRllA and activin A levels in CKD mouse models that produce high-turnover osteopenia caused by increased osteoclast numbers and activated osteoclastic bone resorption compared to that of control mice [23,24,25]. Moreover, the treatment with RAP-011, a ligand trap of ActRllA, has revealed an antiresorptive effect in CKD mouse models [24,25]. Thus, activin A seems to be a positive regulator for osteoclastic development and bone resorption in vivo. However, the molecular mechanisms by which activin A enhances RANKL-induced osteoclastogenesis are yet to be delineated. This review summarizes published data for activin A biology in bone cells and novel agents targeting activin A.

## 2. Regulation of Osteoclastic Development and Bone Resorption

Osteoclasts are large, multinucleated cells with the unique capacity to degrade the organic and inorganic matrices of bone. Osteoclasts are members of the monocyte/macrophage family and, as such share many of the characters of immune cells [26]. Osteoclasts contain some proteins, such as tartrate-resistant acid phosphatase (TRAP), tartrate-resistant trinucleotide phosphatase, carbonic anhydrase II, calcitonin receptors and a few cathepsins (lysosomal proteases), whose main function is bone resorption [27]. Receptor activator of nuclear factor kappa-B (RANK) and RANKL are indispensable for osteoclastogenesis and osteoclastic bone resorption since global RANK or RANKL-deficient mice lack osteoclasts and as result develop severe osteopetrosis [28,29]. RANKL, a type ll membrane protein, belongs to the tumor necrosis factor (TNF) superfamily and contains C-terminal receptor-binding and transmembrane domains, which is mainly produced by bone marrow stromal cells, osteoblasts and activated T-lymphocytes [26,29]. RANK is a transmembrane signaling receptor for RANKL and a member of the tumor necrosis factor receptor (TNFR) superfamily. RANK is mainly expressed on the surface of osteoclast-lineage cells, through the macrophage colony-stimulating factor (M-CSF)-dependent bone marrow macrophages (M-BMMs) stage to mature osteoclasts [30,31]. Expression of RANK is induced by PTH, 1,25-dihydroxyvitamin D3 and prostaglandins [32,33] and these hormones are positively implicated in bone resorption [34]. A study has demonstrated that apoptotic osteocytes produce RANKL stimulating osteoclastogenesis and recruiting osteoclasts to sites of bone remodeling [35]. Osteoprotegerin (OPG), a member of the TNFR superfamily, competes with RANK for RANKL, thus suppressing osteoclast formation and function [36]. OPG is produced by bone marrow stromal cells and osteoblasts regulated by interleukin 1β, TNF-α, TGF-β, estradiol and 17β-estriol. In addition, global OPG-deficient mice develop severe osteoporosis due to increased osteoclast numbers [37,38,39]. 

Early nonspecific differentiation along the osteoclast pathway is dependent on two transcription factors, PU.1 [40] and microphthalmia-associated transcription factor (MITF) [41]. M-CSF is produced by osteoblasts and it stimulates proliferation and survival of the osteoclast precursors [42]. Activation of RANK by RANKL commits M-BMMs to the osteoclast fate and leads to activation of several signaling pathways, such as MAPK and the canonical/noncanonical NF-κB pathways through TNFR associated factor (TRAF6) [43] as well as a Ca^2+^ pathway through the immunoreceptor tyrosine-based activation motif adaptors [44] for immunoglobulin-like receptors, such as *osteoclast*-associated receptor [45] and triggering receptor expressed on myeloid cells 2 [46]. NF-κB pathways contribute to activation of c-Fos and NFATc1, which are essential transcription factors for osteoclastogenesis. NFATc1 is also activated by a Ca^2+^ signal downstream of Ig-like receptors through the tyrosine phosphorylation of signaling molecules [47]. Finally, NFATc1 orchestrates the transcription of osteoclast-specific genes, such as cathepsin K [48], integrin β_3_ [49], dendritic cell-specific transmembrane protein (DC-STAMP) [50], ATPase, H^+^ transporting, lysosomal 38 kDa, V0 subunit d2 (Atp6v0d2) [50], together with PU.1, MITF, NF-κB and c-Fos [43,47]. Osteoclast precursors induced by RANKL express the fusogenic genes, such as DC-STAMP and Atp6v0d2, allowing formation of the multinucleated cell [50]. c-Src [51] and the αvβ3 integrin [52] are required for osteoclast polarization. Once polarized, the osteoclast mobilizes the mineralized component of bone. Bone mobilization is achieved through the acidifying molecules, such as carbonic anhydrase II [53], an electrogenic H^+^ATPase [54] and a charge-coupled Cl^−^ channel [55]. Cathepsin K (Ctsk) is indispensable for bone organic matrix degradation [56]. 

## 3. Activin a Biology in Osteoclastogenesis

In the skeleton, activin A is secreted by osteoblasts and osteoclasts, is abundant in extracellular bone matrix [57,58] and is thought to have fundamental roles in both embryonic skeletal development and postnatal bone homeostasis [9,59]. Activin A has stimulatory effects on several hematopoietic cell lineages, including erythroid [60], megakaryocyte [61,62] and granulocyte-macrophage cells [63]. It is known that monocytes, dendritic cells and macrophages, which are responsible for the differentiation of osteoclasts, produce activin A [64,65,66,67]. We have also detected precursor-inhibin β-A protein expression in M-BMMs and osteoclasts in culture (unpublished data). In culture, several groups including our laboratory have demonstrated that activin A enhances osteoclastogenesis [65,66,68,69,70,71]. In vivo the administration of the soluble extracellular domain of ActRllA fused to a murine IgG2a-Fc (ActRllA-mFc), which is an activin A antagonist, into intact and overiectomized mice (OVX mice: an osteoporosis mouse model) has revealed dual antiresorptive-anabolic effects [72]. ActRllA-mFc treatment increased bone mass and bone strength produced by decreased osteoclastic development and bone resorption plus increased osteoblastic bone formation in these mice. ACE-011, a human ActRllA ligand trap, is also known to have the same dual antiresorptive-anabolic effects in monkeys [73]. ACE-011 stimulated osteoblastic bone formation and inhibited osteoclastic bone resorption in cancellous bone. ACE-011 treatment also resulted in elevation of a marker of osteoblastic bone formation and reduction of markers of osteoclastic bone resorption in healthy postmenopausal women [74]. Consistent with these studies, we have also demonstrated that RAP-011, a mouse ActRllA ligand trap, decreased the number of TRAP-positive osteoclasts and osteoclastic bone resorption in diabetic CKD and AL syndrome CKD mouse models [24,25]. In addition, the treatment stimulated the osteoblastic bone formation in AL syndrome mouse model [25]. Thus, activin A seems to be a positive regulator for osteoclastic development and bone resorption and a negative regulator for osteoblastic bone formation in vivo. However, the molecular mechanisms by which activin A stimulates osteoclastogenesis and bone resorption remain to be elucidated. Sakai and co-investigators have reported that activin A strongly enhances TRAP-positive osteoclastogenesis but not osteoclastic bone resorption, in culture [68]. TRAP-positive osteoclastogenesis were induced from whole bone marrow cells isolated from the mouse long bone tissues in response to 1α, 25-dihydroxyvitamin D_3_ (VD_3_) or PTH with or without activin A. In this study, the treatment of RANKL produced by bone marrow stromal cells with VD_3_ or PTH stimulation and activin A significantly enhanced osteoclastogenesis compared to control cells with VD_3_ or PTH alone. Surprisingly, activin A alone stimulated TRAP-positive osteoclastogenesis compare to control cells without any stimulation. The investigators concluded that in vivo activin A may act as a local factor promoting osteoclastogenesis because bone marrow stromal cells produce activin A [68]. However, these data raised a question of whether activin A is necessary to develop osteoclasts together with RANKL under physiological conditions. Fuller and co-investigators have demonstrated that activin A significantly synergizes with RANKL in osteoclast formation and function in culture that was inhibited by soluble ActRllA treatment, indicating that activin A may be an essential costimulatory for osteoclastogenesis and bone resorption [65]. In this study, it has also shown that activin A acts directly on M-BMMs to enhance osteoclastogenesis. However, it is still unclear that why activin A synergizes with RANKL in osteoclast formation and function. Murase and co-investigators have found for the first time that activin A activates Smad2 and MAPK signaling pathways including ERK 1/2 and p38 in M-BMMs [69]. They concluded that phosphorylation of ERK 1/2, p38 MAPK and Smad2 may be involved in activin A-enhanced osteoclastogenesis induced by RANKL. Smad3 is not important for osteoclastogenesis because mice lacking Smad3 have revealed that osteoclast numbers are normal [75]. We have found another mechanism by which activin A alone stimulates NFκB nuclear translocation in M-BMMs and the event is strongly enhanced by RANKL stimulation, although the treatment of activin A alone failed to induce osteoclasts from M-BMMs [70]. In addition, activin A slightly induced RANK expression at protein levels in the cells. However, activin A is not involved in cell survival during osteoclastogenesis. Therefore, we concluded that activin A brings about a synergistic effect for RANKL-induced osteoclastogenesis through NFκB signaling pathways activated by activin A [70]. However, it remains unclear whether Smad2 associates directly with NFκB during RANKL-induced osteoclastogenesis. More recently, we have demonstrated that NFATc1, Ctsk and integrin β_3_ protein expression levels are remarkably enhanced in RANKL-induced osteoclasts in response to activin A compare to the cells without activin A stimulation (unpublished data). In addition, the chromatin immunoprecipitation (ChIP) assay have shown for the first time that activated-c-Fos and activated-Smad2 are recruited to the NFATc1 promoter in M-BMMs in response to RANKL plus activin A after 24 h. However, activated-Smad2 was not recruited to the promoter in the cells with RANKL alone [24]. Omata and colleagues have identified c-Fos as a critical DNA binding partner of Smad2 during RANKL-induced osteoclastogenesis with TGF-β stimulation by FAIRE (formaldehyde-Assisted Isolation of Regulatory Elements)-seq and ChIP-seq analyses. In addition, they found that RANKL-induced phosphorylation and nuclear translocation of c-Fos in M-BMMs require its association with Smad2. Therefore, they concluded that TGF-β–Smad2 pathway is required for activation of c-Fos and translocation of the Smad2/c-Fos complex into the nucleus during RANKL-induced osteoclastogenesis [76]. We also have confirmed that c-Fos interacts physically with Smad2 by using the immunoprecipitation (IP) assay (unpublished data). More recently, Kajita and colleagues also have demonstrated that activated-c-Fos associates physically with activated-Smad2 so that RANKL-induced osteoclastogenesis and bone resorption are significantly enhanced in response to activin A in culture [77]. On these grounds, we speculated that the recruitment of activated-Smad2 to the NFATc1 promoter by activin A stimulation may cause the enhancement of RANKL-induced osteoclastogenesis. 

Smad2 and Smad3 are 91% identical in amino acid sequence and C-terminally activated-Smad2 and -Smad3 regulate the transcription of many genes in concert with a number of co-associated proteins. However, they have certain differences in biological activity. For instance, while the Smad3 homodimer forms DNA-binding complexes through its MH1 domain without Smad4, Smad2 does not directly bind to DNA due to an additional 30 amino acids, encoded by exon 3. In other words, the exon 3 prevents direct binding of Smad2 to DNA. That’s why Smad2/Smad4 complexes require binding to specific transcription factors to target the complex to DNA [78,79,80,81,82,83,84]. Reportedly, alternative splicing of Smad2 produces the shorter Smad2 (Δexon 3). The shorter Smad2 (Δexon 3) is able to bind to DNA directly and the transcript is strongly expressed in mouse ES cells and embryos at all stages and in adult tissues [85]. However, the role of the shorter Smad2 (Δexon 3) in bone metabolism is completely unclear as well as in other tissues. Smad2 has been demonstrated to associate with many known nuclear proteins including transcription factors, transcriptional co-repressors and transcriptional co-activators [86]. Reportedly, at least, 15 transcription factors, including c-Fos and NFATc1, interact directly with Smad2 that stimulate or suppress their target gene expression [87,88,89,90,91,92,93,94,95,96,97,98,99] (Table 1). Thus, some of transcription factors may be necessary to interact with Smad2 for its nuclear translocation and/or transactivation. In particular, three groups including our laboratory have demonstrated the binding action of Smad2 to c-Fos in osteoclastogenesis [24,76,77]. However, key questions remain regarding that firstly, how activin A-Smad2 axis boosts c-Fos transactivation so that activin A enhances RANKL-induced osteoclastogenesis? And secondly, Smad2 is essential factor for osteoclastogenesis physiologically? The critical role of c-Fos in osteoclastogenesis has been extensively studied. Mice lacking c-Fos exhibit osteopetrosis because of deficiency in osteoclastogenesis [100]. The osteopetrotic phenotype of c-Fos-deficient mice was rescued by transgenic overexpression of c-Fos or Fra-1 [101]. In addition, c-Fos binds to and cooperates with NFATc1 to promote osteoclastogenesis [102]. Thus, c-Fos is an extremely critical transcription factor for the differentiation from M-BMMs to mature osteoclasts. On the other hand, we recently found that in culture RANKL induces osteoclastogenesis in Smad2 deficiency similar to control cells. However, activin A failed to enhance RANKL-induced osteoclastogenesis in Smad2 deficiency (unpublished data), indicating that Smad2 may be not necessary for osteoclastogenesis in physiological conditions. Yet, we need to confirm the role of Smad2 for osteoclastic development and function in mice. In our ChIP assay, RANKL alone recruited only activated-c-Fos to the NFATc1 promoter. Interestingly, RANKL and activin A treatment recruited both activated-c-Fos and activated-Smad2 to the NFATc1 promoter, although activin A alone failed to recruit both of them to the NFATc1 promoter. In addition, our quantitative RT-PCR analysis for the ChIP assay has shown that the combination of RANKL and activin A stimulation strongly stimulated activated-c-Fos DNA binding to the NFATc1 promoter compared to RANKL alone. In contrast, this effect was reduced in Smad2-deficient M-BMMs. Moreover, the treatment of RANKL plus activin A significantly stimulated activated-c-Fos nuclear translocation compared to that of nuclear translocation with RANKL alone in M-BMMs (unpublished data). These results strongly suggest that activin A treatment may enhance protein-protein interactions, such as c-Fos and Smad2 and accelerate the nuclear translocation of the complex so that NFATc1 protein expression levels are strongly elevated in RANKL-induced osteoclastogenesis in response to activin A.

**Figure 1:**
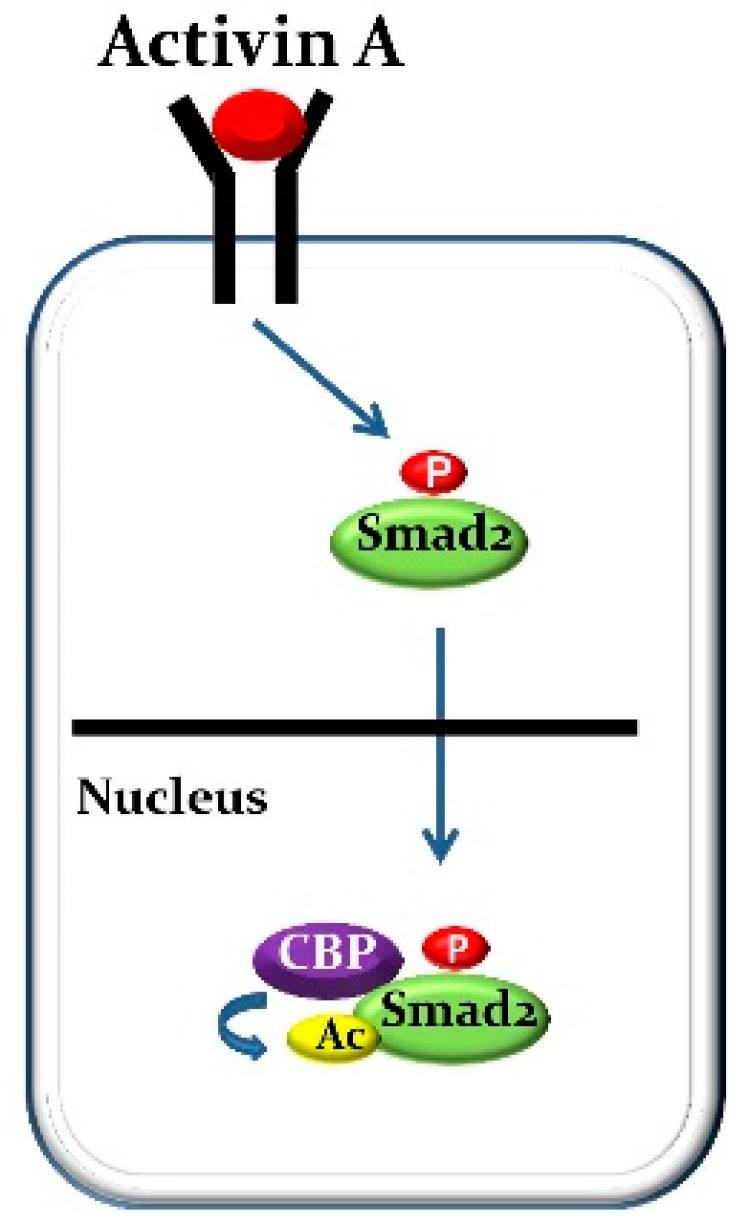
**Activation of activin A signaling stimulates Smad2 acetylation.** Smad2 is strongly acetylated by activin A stimulation in M-BMMs because CBP is localized only in nucleus and its association with Smad2 increases upon nuclear accumulation of Smad2 as a result of activin A treatment.

**Figure 2:**
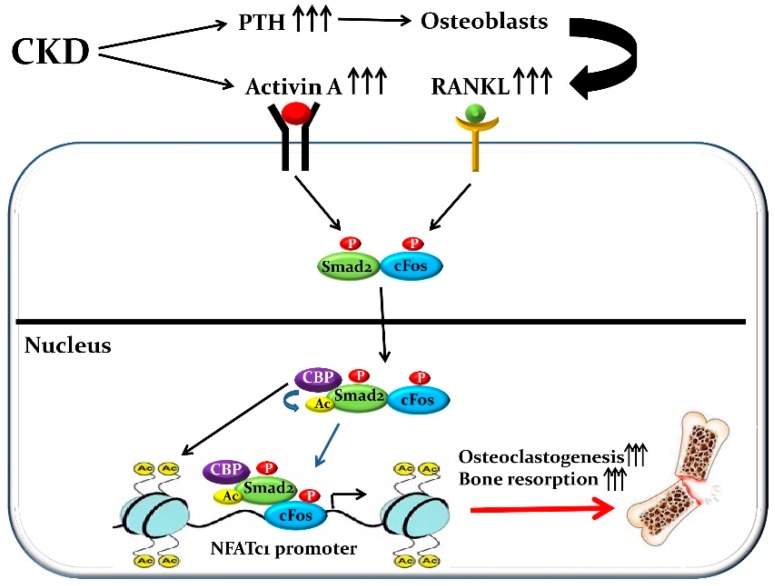
**Activation of activin A signaling stimulates RANKL-induced osteoclast development and function in CKD.** The nuclear translocation of c-Fos and the DNA binding of activated-c-Fos to the NFATc1 promoter is induced by RANKL alone without Smad2 for osteoclast formation and function. In contrast, the combination of RANKL and activin A treatment induces a complex composed of activated-c-Fos and activated-Smad2 and stimulates the nuclear translocation of the complex so that the DNA binding of activated-c-Fos to the NFATc1 promoter is strongly boosted by acetylated-Smad2 caused by CBP.

Non-histone protein acetylation has been shown to influence a diverse array of biochemical properties including protein-protein interactions, DNA binding activity, protein stability and intracellular localization [103]. It has been demonstrated that Smad2 but not Smad3, can be acetylated by p300/CBP (CREB-binding protein), which are co-activator with histone acetyltransferase activity [104] and this acetylation event plays a role in stimulating the nuclear translocation of Smad2 upon TGF-β treatment [105]. Our IP assay also has shown that CBP physically binds to Smad2 but not c-Fos. In addition, Smad2 was strongly acetylated by activin A stimulation in M-BMMs (unpublished data). This result was expected because CBP is localized only in nucleus and its association with Smad2 increases upon nuclear accumulation of Smad2 as a result of activin A treatment (Figure 1). On these grounds, we conclude that the nuclear translocation of c-Fos and the DNA binding of activated-c-Fos to the NFATc1 promoter can be induced by RANKL alone without Smad2 for osteoclast formation and function. However, the combination of RANKL and activin A treatment induces a complex composed of activated-c-Fos and activated-Smad2 and stimulates the nuclear translocation of the complex so that the DNA binding of activated-c-Fos to the NFATc1 promoter may be strongly boosted by acetylated-Smad2 caused by CBP. That’s why RANKL-induced osteoclastogenesis and osteoclastic bone resorption may be strongly activated by activin A stimulation (Figure 2).

## 4. Regulation of Osteogenesis and Bone Formation

Osteoblasts are bone-forming cells and produce a unique combination of extracellular proteins, such as alkaline phosphatase (ALP), type 1 collagen and osteocalcin, which have an essential role in bone mineralization [106]. Osteoblasts can differentiate from bone marrow mesenchymal progenitor cells through two processes, intramembranous or endochondral ossification. Bone marrow mesenchymal progenitor cells directly differentiate into osteoblasts during intramembranous ossification. In contrast, bone marrow mesenchymal progenitor cells give rise to chondrocytes and perichondrial cells and the latter cells differentiate into osteoblasts during endochondral ossification [107]. Some of osteoblasts turn into osteocytes upon being entombed in the bone matrix and the rest cells are eliminated by apoptosis or turn into inactive bone-lining cells [106]. Some of transcription factors are involved in osteoblastogenesis [106]. Runt-related transcription factor 2 (Runx2) is indispensable for osteoblastogenesis during both intramembranous or endochondral ossification. Mice lacking Runx2 produced a complete lack of osteoblasts [108,109]. A zinc-finger transcription factor, osterix (Osx), also plays critical roles during osteoblastogenesis. The deletion of Osx resulted in complete absence of osteoblasts in mouse embryos, although Runx2 expression was relatively normal. In contrast, Osx expression was eliminated in Runx2-deficient mice, indicating that Osx functions down-stream of Runx2 during osteoblastogenesis [110].

## 5. Activin a Biology in Osteoblast Development and Function

Smad-dependent TGF-β signaling stimulates proliferation, chemotaxis and early differentiation of osteoblasts from mesenchymal stem cells to immature osteoblasts. However, it suppresses osteoblast maturation, mineralization and transition into osteocyte [75,111,112]. Activated-Smad3 with TGF-β treatment recruits class ll histone deacetylases (HDACs), such as HDAC4 and HDAC5, to suppress Runx2 functions and TGF-β is unable to suppress osteoblastogenesis in Smad3 deficiency [75,112,113,114,115]. Thus, the inhibitory effects on TGF-β for mature osteoblasts and bone mineralization has been well-established. In contrast, the roles of activin A for osteoblast differentiation and function are not well-understood, even though many have reported the possibly inhibitory effects on activin A for osteoblastogenesis and bone mineralization [73,116,117,118,119,120,121,122,123]. As described, ActRllA-mFc, ACE-011 and RAP-011 have shown the anabolic effects on OVX mice, postmenopausal women and CKD mouse models, respectively [24,25,72,74], indicating that activin A should have the negative effects on osteoblastogenesis and bone mineralization. Yet, the molecular mechanisms have been remained unclear yet. For instance, like TGF-β, do Smad-dependent activin A signaling pathways negatively regulates Runx2 functions so that osteoblastogenesis is impaired? Further studies are needed to elaborate the role of activin A in osteoblast function.

## 6. The Mechanisms of the Medial Vascular Calcification Caused by CKD

Cardiovascular disease is the main cause of morbidity and mortality in patients with CKD. Vascular calcification is an independent predictor for the mortality and morbidity of patients with CKD and vascular calcification caused by CKD is more frequent than in people of the same age and gender without CKD [124,125]. This forms the paradox of inhibition of skeletal mineralization and stimulation of heterotopic mineralization in CKD. Vascular calcification is classified into two major types, intimal and medial and arterial medial calcification is prevalent in aging and patients with CKD and diabetes [126]. Medial vascular calcification occurs in the absence of inflammation, is the earliest type of vascular calcification found in children with CKD and is considered a hallmark of CKD-MBD in adults [124,125,127]. It has been widely accepted that intimal and medial vascular calcification are closely associated with increased cardiovascular mortality. Arterial intimal calcification is associated with plaque rupture and myocardial infarction. In contrast, arterial medial calcification leads to vessel stiffening, increased pulse-wave velocity, reduced cardiac perfusion, and, ultimately, left ventricular hypertrophy and heart failure. The most important thing is that heart failure is a predominant cardiovascular cause of death in patients with CKD [124,127]. Therefore, understanding the cellular and molecular mechanisms mediating medial vascular calcification is critical for improved therapeutics for high-risk patients with CKD.

Vascular calcification is a highly regulated process that resembles skeletal bone formation. Some of osteogenesis-related transcription factors, such as Msh homeobox 2 (Msx2), Osx, Runx2 and activating transcription factor 4 (ATF4), are expressed in both calcified medial arterial layers and atherosclerotic plaques [126,128,129]. However, the molecular mechanisms by which osteogenic transcription factors promote osteogenic differentiation of vascular smooth muscle cells (VSMCs) or suppress VSMC differentiation still remain unclear. Tanaka and co-investigators have demonstrated for the first time in vitro that forced expression of Runx2 decreases the expression of VSMC genes and promotes osteogenic gene expression, whereas the reduction of Runx2 expression by small interfering RNA (siRNA) stimulates VSMC differentiation in human aortic VSMCs because Runx2 binds to serum response factor (SRF), which is a transcription factor regulating VSMC differentiation together with myocardin [130] and interferes with the formation of the SRF/myocardin ternary complex. Thus, Runx2 inhibits SRF-dependent transcription, as a corepressor independent of its DNA binding [131]. Consistent with in vitro studies, high-fat diet induced-intimal vascular calcification was dramatically improved in VSMC-specific Runx2-deficient mice lacking apolipoprotein E (ApoE) due to decreased ALP expression and aortic calcium contents [126]. Vitamin-D-induced medial vascular calcification was also significantly improved in VSMC-specific Runx2-deficient mice [132]. In contrast, VSMC-specific overexpression of Runx2 in transgenic mice produced medial fibrosis and aortic stiffening due to increased type 1 collagen a1 (Col1a1) and a2 (Col1a2). However, the mice failed to induce medial vascular calcification. In this study, NFκB RelA (p65) binding site was identified within the human Runx2 promoter. In fact, RelA gene silencing by siRNA completely knocked-down the high glucose-induced Runx2, Col1a1 and Col1a2 gene expression in human aortic VSMCs [133]. On the other hand, there is no report whether CKD-induced medial vascular calcification is improved or induced in Runx2 loss-of-function or gain-of-function in mice, respectively.

ATF4 has also been implicated in the onset of intimal and medial vascular calcification. ATF4 plays critical roles in the late stage of osteoblastogenesis and directly induces osteocalcin and Osx [134,135]. CKD-induced medial vascular calcification was dramatically improved in global ATF4-haplodeficient mice with DBA/2J background, which are susceptible to medial vascular calcification with high phosphorus diet. In contrast, VSMC-specific overexpression of ATF4 in transgenic mice with DBA/2J background strongly induced CKD-induced medial vascular calcification compare to control mice with CKD. In addition, the transgenic mice with ApoE deficiency significantly stimulated high-fat diet induced-intimal vascular calcification. A new finding in this study is that ATF4 transcriptionally stimulates expression of type lll sodium-dependent phosphate cotransporters, Pit1 and Pit2, by interacting with CCAAT/enhancer-binding protein beta [129]. Runx2 is expressed in the early stage of osteoblastogenesis and ATF4 is expressed in the late stage of osteoblastogenesis. Runx2 or ATF4 triggers Osx gene expression directly. However, no Runx2 stimulates ATF4 gene expression [105,135]. Therefore, one question remains unanswered that why loss-of-function of ATF4 in VSMCs prevented CKD-induced medial vascular calcification in spite of Runx2 expression remains in ATF4-deficient VSMCs [129]? Why Vitamin-D-induced medial vascular calcification is improved in VSMC-specific Runx2-deficient mice in spite of ATF4 is expressed in Runx2-deficient VSMCs [132]? Further investigation regarding osteoblastic transition in CKD induced vascular calcification is needed. 

## 7. Activin a Biology in CKD-Induced Medial Vascular Calcification

TGF-β1 was shown to play critical roles in vascular calcification. TGF-β1 is expressed in calcified aortic valves and is involved in osteoblastic transition of VSMCs [136,137]. In contrast, there is no report whether activin A is involved in the making of CKD-induced medial vascular calcification except our recent reports. We have demonstrated for the first time that systemic activin A is elevated and RAP-011 treatment improves CKD-MBD in our CKD mouse models [23,24,25]. The treatment improved ActRllA signaling in aortas so that contractile VSMCs-specific protein expression is increased and osteoblastic transition is decreased. Therefore, CKD-induced vascular calcification was improved, indicating that activin A-dependent Smad signals may be implicated in the formation of vascular calcification [23,24,25].

## 8. Conclusions and Future Perspectives

Although there is no report that systemic activin A is elevated in patients with CKD, we strongly indicate through a series of our current studies [23,24,25] that activated-systemic ActRllA signaling may be implicated in the onset and the progression of CKD-MBD. Reportedly, systemic activin A levels are increased in postmenopausal women, aging and patients with type 2 diabetes mellitus [138,139,140,141,142]. Diabetes mellitus is closely implicated in CKD-MBD. The prevalence of vascular calcification, which becomes higher in diabetic CKD patients than in non-CKD counterparts, increases cardiovascular mortality in diabetic patients [143]. That’s why it is strongly suggested that systemic activin A levels should be elevated in diabetic CKD patients that produces vascular calcification and ROD. In addition, CKD-MBD has phenotypic similarities that reflect premature aging, such as medial vascular calcification and osteoporosis [144]. For instance, even CKD in children produce medial vascular calcification, which is a hallmark of vascular aging. Moreover, emerging evidence has shown that CKD may cause DNA damage, which is a hallmark of cellular senescence and DNA damage up-regulates inhibin β-A gene expression [145,146]. In the skeleton, the treatment of ACE-011, an activin antagonist, increased bone formation and decreased bone resorption that improved cancellous but not cortical, bone volume, microarchitecture and mechanical strength in primates [73]. In a single-dose phase I study in healthy postmenopausal women, ACE-011 treatment caused a rapid, sustained and dose-dependent increase of bone formation markers and a decrease of bone resorption markers [74]. However, there are currently no further clinical studies of this compound being conducted in patients with osteoporosis. On these grounds, we speculate that activin A is implicated in not only CKD-MBD but also premature aging because the some of the phenotypes of CKD-MBD overlap with that of premature aging, such as medial vascular calcification and osteoporosis. However, it remains unclear whether activin A activates or suppresses age-promoting mechanisms and anti-aging pathways. In any case, increased-systemic activin A can be a biomarker of CKD-MBD that can be targeted for CKD-MBD prevention and therapy.

## Figures and Tables

**Table 1:** Smad2 Nuclear Interacting Transcription Factors.

Transcription Factors: Official Symbol/(Official Full Name)	References
Fos (FBJ osteosarcoma oncogene)	[24,76,77]
Myc (MYC proto-oncogene, bHLH transcription factor)	[87]
Evi-1 (ecotropic viral integration site 1)	[88]
Foxh1 (forkhead box H1)	[89,90]
Gli3 (GLI-Kruppel family member GLI3)	[91]
Hoxa13 (homeobox A13)	[92]
Lemd3 (LEM domain containing 3)	[93]
Mef2a (myocyte enhancer factor 2A)	[94]
Runx2 (runt related transcription factor 2)	[95]
Sp1 (trans-acting transcription factor 1)	[96]
Zeb1 (zinc finger E-box binding homeobox 1)	[97]
